# Terminal investment induced by a bacteriophage in a rhizosphere bacterium

**DOI:** 10.12688/f1000research.1-21.v2

**Published:** 2013-05-20

**Authors:** Timothée Poisot, Thomas Bell, Esteban Martinez, Claire Gougat-Barbera, Michael E Hochberg

**Affiliations:** 1 Université Montpellier II, Institut des Sciences de l’Evolution, Montpellier, France; 2 Département de Biologie, Chimie et Géographie, Université du Québec à Rimouski, Rimouski, QC, G5L 3A1, Canada; 3 Québec Centre for Biodiversity Sciences, Stewart Biological Sciences Building, Montréal, QC, H3A 1B1, Canada; 4 Department of Life Sciences, Imperial College London, Silwood Park Campus, Ascot, Berkshire, SL5 7PY, UK; 5 Santa Fe Institute, Santa Fe, NM, 87501, USA

## Abstract

Despite knowledge about microbial responses to abiotic stress, few studies have investigated stress responses to antagonistic species, such as competitors, predators and pathogens. While it is often assumed that interacting populations of bacteria and phage will coevolve resistance and exploitation strategies, an alternative is that individual bacteria tolerate or evade phage predation through inducible responses to phage presence. Using the microbial model *Pseudomonas fluorescens* SBW25 and its lytic DNA phage SBW25Φ2, we demonstrate the existence of an inducible response in the form of a transient increase in population growth rate, and found that the response was induced by phage binding. This response was accompanied by a decrease in bacterial cell size, which we propose to be an associated cost. We discuss these results in the context of bacterial ecology and phage-bacteria co-evolution.

## Introduction

Pathogens are ubiquitous in natural communities ^1^ and the antagonistic interactions they establish with their hosts are recognized as one of the main drivers of evolutionary diversification ^2, 3^. Hosts can reduce the impact of pathogens through three non-mutually exclusive processes ^4^: (i) avoidance of either infected individuals, habitats where the pathogen is prevalent, or of the pathogen itself ^5^, (ii) resistance to the actual infection process or post-infection immune defences ^6^, and (iii) tolerance ^7^. Research on these responses has generally focused on animal and plant models, but there is growing appreciation that microbes, particularly bacteria, can exhibit similar responses. For instance, bacteria can be selected for heightened levels of genetic resistance towards infection by pathogens ^8– 10^. On the other hand, although bacteria are known to display plastic responses to various types of environmental stresses ^11, 12^ and to competition ^13^, it is unknown whether they can do so when faced with natural enemies such as bacteriophages.

Plastic responses are an adaptive phenotypic change following an environmental stimulus, occurring without a concurrent change in the genotype ^14^. They may involve behavioural, physiological or phenological changes ^15, 16^, and be triggered by direct or indirect contact with the stimulus ^17^ or through communication with neighbouring organisms ^18^. Phenotypic plasticity is considered to be a genetic adaptation to variable environments, but given the diversity of associated mechanisms and behaviours, it is not known to what extent different stimuli translate into different responses ^15, 19^.

Individual-level interactions between bacteria and phage may be conducive to induced responses. The first step of bacteriophage infection is the binding of phage proteins to bacterial surface proteins ^20^, which then triggers conformational changes to both proteins ^21^. Surface proteins used by the bacterium for signal transduction are known to be targets of bacteriophage adsorption ^22^ and as such could trigger a response when bacteriophage binding is detected. Such a response would allow a bacterium to react to the pathogen and to eventually either evade or reduce the effects of the infection. Lytic phages are prime candidates for organisms against which bacteria may have evolved a stress response, because they typically interact with their host over short timescales, and death is inevitable once the phage has injected its DNA into a sensitive bacterial cell.

In addition, bacteriophages are widely distributed in the environment ^20^ and interact with their hosts over relatively small spatial scales ^23^ and throughout most of the year ^24, 25^. This could select for the expression of induced structural, physiological or behavioural responses to different enemies. Also, bacteria employ signalling pathways and have a known ability to communicate within populations ^26^. Such pathways could induce and synchronise inducible responses before predators and pathogens are encountered, or at least before they have spread through the population, or before the point beyond which cell death is certain. All of these factors suggest that plastic stress responses to phage should be a common feature of bacterial cells and that such responses would have important repercussions for ecological and evolutionary interactions between phage and bacterial populations. Although molecular responses of bacteria to bacteriophages have been characterized ^27^, the behavioral, ecological, and selective consequences of such responses are not known.

Here we demonstrate that when confronted with phage, bacteria express transient increases in division rate at a cost to individual biomass accumulation ^28^. Specifically, we employ the rhizosphere bacterium *Pseudomonas fluorescens* SBW25 ^29^ to investigate how its population growth rate is affected by exposure to inactivated populations of is lytic bacteriophage SBW25Φ2 ^30^. We find that bacteria exposed to inactivated phage increase their fission rate nearly two-fold at 24 hours post-exposure. This is followed by a continual decrease in fission rate relative to the control. We also show that bacteria exposed to inactivated phage were smaller in size compared to controls. By the end of the experiment, bacteria regained their original growth rate, but not their original size, which implies differences in energy allocation constraints between these two life-history traits. All of these effects were enhanced as the density of inactivated phage was increased. The results are consistent with a behavioural strategy that increases allocation to reproduction under stressful conditions (i.e., “terminal investment”). Terminal investment is well characterised for other host-parasite associations ^31^, but to our knowledge has not previously been observed in bacteria subject to phage infection.

## Results

**Figure 1.**
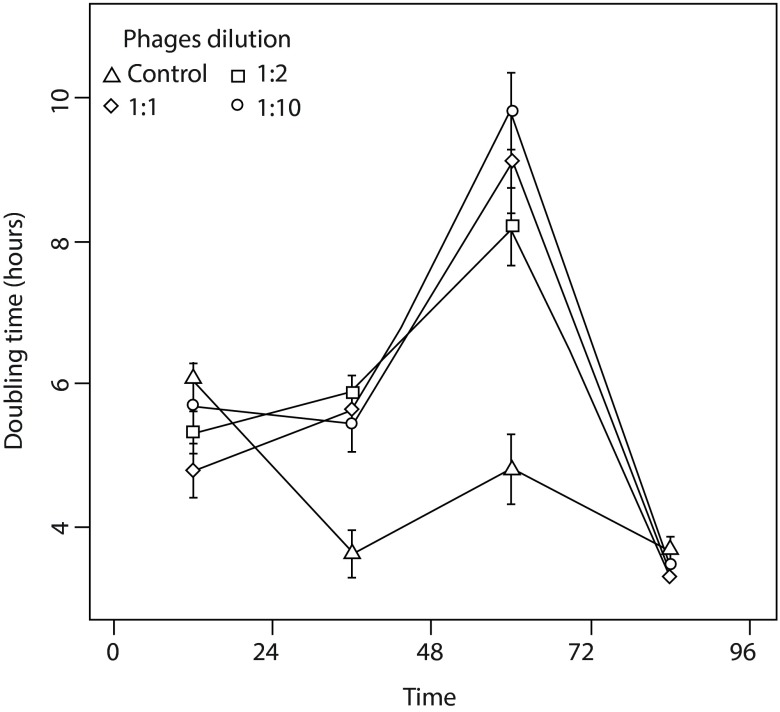
Maximum doubling time (in hours) of biomass produced by bacteria exposed to different concentrations of UV-inactivated phage. This was measured for four consecutive days following four hours exposure. Bacteria exposed to phage grew significantly faster than controls over the first day, and then expressed an apparent cost in terms of smaller cell size that attenuated by the fourth day. Central points are the means of 12 replicates, and the bars are standard errors.

Bacteria exposed to UV-inactivated phage display a statistically significant higher growth rate over the first 24 hours post-exposure than non-phage controls (Kruskal-Wallis, df = 3, P = 0.006; Figure 1). After this period, the estimated doubling time of exposed bacteria increased (i.e., their populations grew slower), and did so for the next 48 hours. This decrease in growth rate compared to controls is suggestive of a cost to the higher fission rate observed over the first 24 hours ( Figure 1). During the fourth day post-exposure, control and treatment bacteria showed no significant differences in doubling time (KW, df = 3, P > 0.05). That exposed bacteria returned to their ancestral growth rate suggests that the response over the first 24 hours was due to phenotypic plasticity and not selection on faster growing genotypes. There was a marginally significant effect on population growth for bacteria exposed to different phage concentrations (KW, df = 2, P < 0.02), suggesting that the encounter rate between bacteria and phage is important in determining the population-level strength of the fission response.

Bacterial doubling time, expressed in hours, as a function of the treatment Day: Day of the measure (maximal doubling time over 24 hours growth period) Phages: Experimental treatment (see the paper) dt: Doubling time, in hours

**Figure 2.**
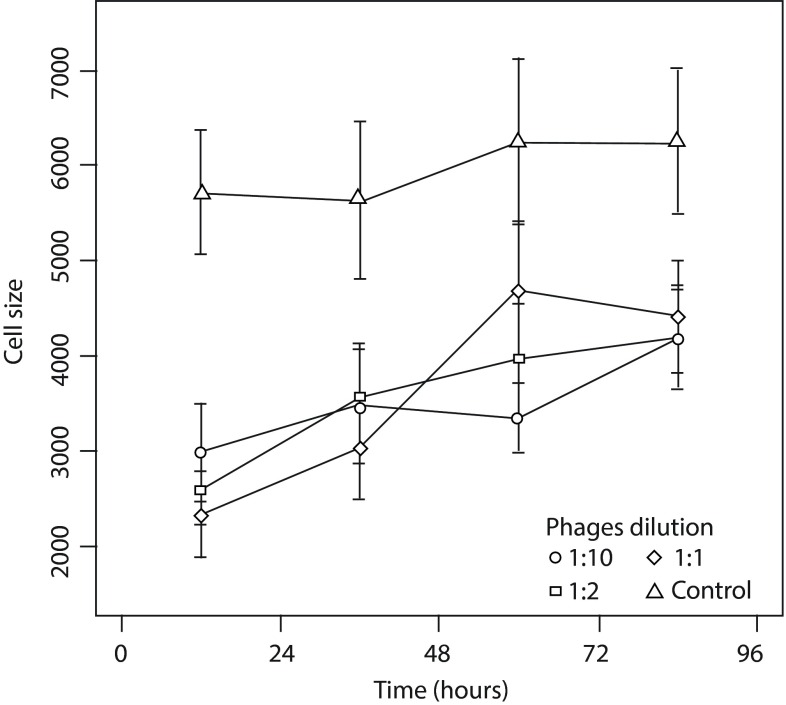
Mean bacterial cell size (forward scatter parameter) exposed to different concentrations of UV-inactivated phage, as per the method in Figure 1. Bacteria exposed to phage at different concentrations do not significantly differ in size. Points and bars are the same as in Figure 1.

We hypothesized that faster doubling times would come at a cost to cell size, since cells would have less time to metabolize and convert absorbed nutrients into cell structure twenty-four hours post-exposure, we found that phage-treated bacteria were two to three times smaller (as measured by mean cellular width) than the control (KW, df = 3, P < 0.0001; Figure 2). This difference in size gradually decreased over the following 3 days, but in contrast to growth rate ( Figure 1), bacteria did not attain their ancestral cell size by the end of the experiment ( Figure 2). Analyses of the distribution of several flow cytometry profiles showed that a difference in cell shape is unlikely to explain this result (see Data File below). Namely, whereas the bacterial populations differed with regards the *side scatter* parameter, *forward scatter* showed no change in its distribution. This implies that bacterial shape remained unchanged throughout the experiment, and indeed, additional observations using a transmission electron microscope showed that the cells remained rod-shaped for all treatments.

We did not observe any difference in the impact of live phage on bacterial populations exposed to the different treatments (KW, df = 2, P = 0.153), suggesting that the inducible response does not alter bacteria resistance to phage predation.

Raw flow cytometry data Day: Day of the measure Treatment: Experimental treatment Other columns are the parameters for each object (bacteria)

## Discussion

Our experiments reveal a previously unexplored behavioural response to bacteriophage predation: phage induce bacteria to reproduce earlier in their cell cycle. We hypothesize that this response increases the survival chances of bacterial progeny under natural conditions and demonstrate that this behaviour comes at a fitness cost of reduced size of daughter cells. Our experiments with UV-inactivated phage further demonstrate that this response is specifically due to phage binding. An alternative explanation is that phage binding decreases resource uptake by bacterial cells. However, this seems unlikely in our experiment. Because bacteria were exposed to inactivated phages only, the total number of viral particles is predicted to stay constant (or possibly degrade) throughout the experiment. When bacteria divide, the number of phages bound to a daughter cell should be roughly half the number on the mother cell; thus, the number of bound phages per cell will decrease exponentially with cell divisions. Using the density of phages and bacteria employed in our experiment, we predict that there will be, on average, less than one phage individual per bacterial cell after 9 to 10 cell divisions, which based on the mean doubling time presented in Figure 1, is reached in the first 48 hours of the experiment. Our results can explain previous observations on phage-associated increases in population size in *P. fluorescens* ^32^. Specifically, we predict that a significant number of phage in the experiments of Gomez and Buckling ^32^ did not kill their bacterial hosts before some of the latter were able to accelerate their cell cycle and produce daughter cells. Furthermore, our results support and extend both theoretical ^33^ and empirical ^34, 35^ predictions that victims may lessen the fitness impact of their natural enemies through early reproduction, to cases where phenotypic responses are plastic and temporary. Increased allocation to reproduction in stressful environments–termed “fecundity compensation” or “terminal investment” ^31^–although never studied in bacteria-phage associations to our knowledge–has been extensively studied for other host-parasite (or organism-stressor) interactions. Terminal investment is characterized by increased reproductive rate or the earlier onset of reproduction, if the prospect of future reproduction is low ^36^. Examples of such responses include faster host maturation ^37^, increased oviposition rate ^38^, and the modification of traits involved in the onset of reproduction ^39, 40^. This response is expected to result in smaller individual size, because energy allocated to growth is directed to reproduction when the stressor is present.

Phenotypically plastic responses are important in that they allow individuals to cope with environmental change during their lifetimes ^41^. As such, plasticity is expected to be favoured in variable environments when the costs of induction and phenotypic change compensate for probabilistic (expected) fitness loss ^42^. Although it is difficult to generalize about constitutive costs of resistance across biological systems ^43, 44^, limited evidence suggests that genetically evolved, constitutive resistance in bacteria to their lytic phage could have costs of as much as 5–10% to relative fitness ^45^.

We employed inactivated bacteriophages to evaluate how phage contact with the bacterial outer membrane mediates bacterial responses. Bacteria could be selected to exhibit an escape response in several, non-mutually exclusive ways. First, non-virulent phage may signal the presence of virulent phage in the local environment (i.e., the bacterium does not perish following initial phage contact). Senescent (inactive) phage are present in natural environments ^25^, and many phages bind to outer membrane proteins without being infective (e.g. the bacterium is resistant; ^45^). Moreover, it is possible that phage could detach if they sense the host to be unsuitable ^46^. Second, when phage infect the bacterium there may be a ‘race’ between the time it takes a bacterial cell to divide (and potentially survive) and the point of no recovery associated with the maturation of phage progeny and bacterial cell lysis. Third, the response may be a consequence of lysogens competing with lytic phages for host exploitation; the latter could benefit from early host reproduction in the presence of lytic competitors. However, sequencing of the *P. fluorescens* SBW25 genome revealed a low abundance of prophage-like regions ^47^.

We were not able to determine whether the bacteria or the phage benefit from faster bacterial reproduction, and the literature reports effects both of facilitation and decrease in host metabolism upon infection ^48^. Previous theoretical work suggests that phage productivity increases in bacteria with short life-cycles ^49^. This is supported by recent empirical study employing the same strain of *P. fluorescens* ^50^. Assuming that the physiological mechanisms involved in fission rate increases are the same in the two experiments, this suggests that rapid multiplication is not adaptive for the bacterium, and indeed we report no advantage of being exposed to inactived phage in terms of a lessened population impact during live phage exposure. Upon exposure to phage, bacteria reproduce faster, but experience a persistent reduction in individual size. Smaller cells have less surface area, and assuming that the density of receptor proteins does not change with cell size, this suggests that they will have lower encounter rates with phage. One possibility is that cell division allows bacterial cells to concentrate phage in one of the daughter cells ^51, 52^, resulting in some progeny managing to escape the pathogen. Future studies should therefore focus on the possible adaptive nature of this response for both bacterium and phage, by investigating in greater depth how it affects the mechanisms of infection, recovery, and resistance.

## Methods

### Bacteria cultures

Ancestral *Pseudomonas fluorescens* SBW25 ^29^ were inoculated into 30 ml microcosms containing 6 mL of King’s B medium (KB), and allowed to grow under alternating rotational agitation (200 rpm for 1 minute every 30 minutes). Every 48 h following plating on solid agar, 10 CFU of the *smooth* morphotype were transferred into fresh KB medium. After 10 transfers, the culture was composed of *smooth* morphotypes only. We continued this selection procedure for another 10 transfers and then arbitrarily isolated a single CFU, which was used for all experiments described below. Experiments were conducted at 28°C in KB medium under constant rotational agitation (200 rpm).

### Phage cultures

**Supplementary Figure S1.**
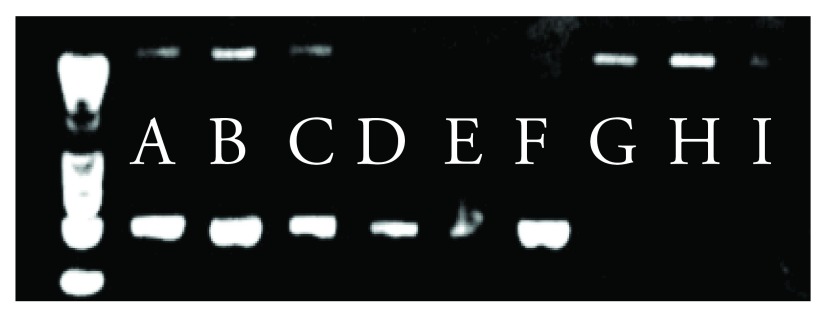
Sample gel obtained on 9 total DNA extractions (A–C: bacteria and page, D–F: phage only, following extraction as explained in text, G-I: bacteria following exposure to inactivated phage, whose DNA was extracted after removal of inactivated phages). The primers TPV1f and TPV1r yield a 1200 bp amplicon in the bacteria, and a 500 bp amplicon in phages. Our seperation method for bacteria and phage was complete, since only DNA of the intended organism was found in any given sample.

**Supplementary Figure S2.**
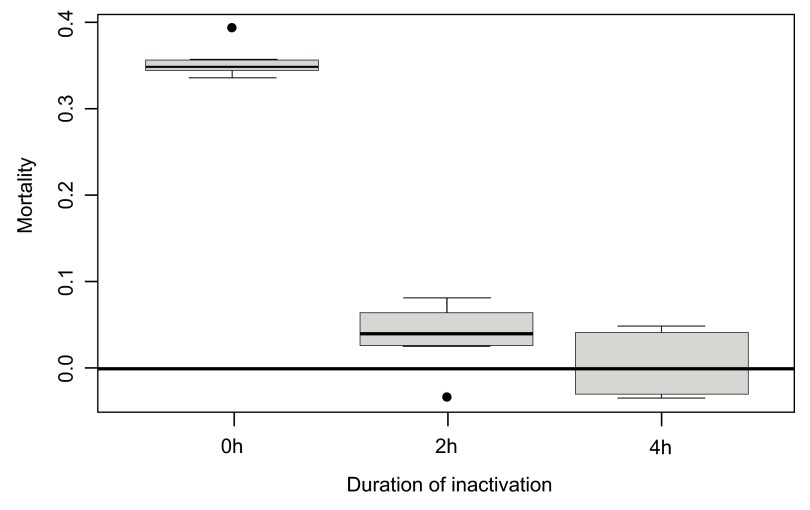
Bacterial mortality as a function of phage inactivation. Because bacteria do not grow at 4°C, we can directly measure phage-induced mortality. After 4 hours of exposure to UV, we observed that phages do not introduce significant mortality in the bacterial population. Kruksal-Wallis test (df = 2, p = 0.02) reveals differences between treatments, with 0h and 2h being significantly different (t-test, df = 7, p < 10 ^-5^), 0h/4h being significantly different (t-test, df = 8, p < 10 ^-5^), and 2h and 4h being similar (t-test, p = 0.16) - all p-values were Bonferroni-corrected to account for multiple testing. Similarly, 2h and 4h are not significantly different from 0 (p-values of -5 -5 0.14 and 0.89 respectively, after correction).

We grew an arbitrarily selected clone of the ancestral phage SBW25Φ2 ^30^ on an exponentially growing culture of fixed *smooth* *P. fluorescens* SBW25 in 3 mL of KB for 48 hours. This resulted in a culture containing approximately 10 ^8^ phage per ml. The sample was then centrifuged for 3 minutes at 8000 rpm in a 1.5 ml Eppendorf tube, and the pellet discarded. Centrifugation was repeated three times to ensure all bacteria were removed (see Supplementary Figure S1). Phages were then isolated by centrifuging the remaining supernatant for 8 minutes at 13000 rpm, and inoculating the pellet into fresh KB medium. The sample was thoroughly vortexed and exposed to UV light (Model 4.LC, Vilber Lourmat, Deutschland, 254 nm wavelength) at 5 cm distance for 4 hours. Extensive pilot studies demonstrated that this method was sufficient to kill all phage (see Supplementary Figure S2).

### Preliminary tests

We conducted a series of preliminary tests to verify how UV-inactivated phage affected bacterial hosts. First, observations under a transmission electron microscope showed that UV-inactivated phage were still intact and able to bind to their bacterial hosts. Second, we checked that bound UV-inactivated phage did not introduce phage DNA into the bacteria. This was done by inoculating 1 ml of UV-inactivated phage into 6 overnight bacterial cultures. Inactivated phage were allowed 4 h to attach to the bacterial outer membrane. We separated phage and bacterial fractions by filtration using a 0.2 µm filter. We then conducted a full DNA extraction (WholeBlood NucleoSpin DNA extraction kit, Macherey-Nagel) of the filter. PCR was done using *TPV1f* (GATGTGAGAAAGCGATACACGG) and *TPV1r* (GAGAGAAGCGGGAGAGTGAA) sequences developed for this study, which selectively amplify a 550 bp fragment of the phage DNA and a 1200 bp fragment of the bacterial DNA (see Supplementary Figure 1 for detailed protocols). We did not find any evidence that UV-inactivated phage was present in samples putatively containing bacteria only, thus confirming that (i) the DNA of inactivated phage was not incorporated in the bacterial cell and (ii) our centrifugation method removed both bound and unbound phage. Observations of *c.* 50 cells using TEM (Zeis EM10) showed no bound phages after the centrifugation treatment.

### Experiments using UV-inactivated phage

We conducted an experiment to understand how UV-inactivated phage affected bacterial behaviour. Fixed SBW25 bacteria of the *smooth* morphotype were first cultivated in 6 ml KB in 30 mL universal glass vials. 20 µL of exponentially growing bacteria ( *c* 10 ^4^ bacterial cells) were transferred into fresh KB medium with either no phage or UV-inactivated phage at ratios of 1:10, 1:2, and 1:1 (corresponding to approximately 10 ^6^, 5×10 ^6^, and 10 ^7^ phage per ml), and then allowed to interact for 4 hours under alternating shaking (200 rpm for 1 minute every 30 minutes). KB medium containing UV-inactivated phages was obtained through centrifugation of inactivated phage, which were further added into pure KB, so that the medium used in the treatments only differs from the control by the presence of phages. Bacteria were then separated from bound phage by centrifuging (see above) and placed in fresh KB medium. 1% of each population was transferred every 24 hours into new KB medium. Each of the 4 treatments was replicated 6 times and arranged arbitrarily in a rack for incubation.

### Measures

Biomass doubling time (used as a proxy for population fitness) was measured in a Fluostar Optima spectrophotometer (28°C, constant agitation, 250 measures at 650 nm over 24 hours) each day, using the following formula:

(1)      D _t_ = [∆t ln(2)]/[ln(N*) – ln(N _0_)]

where N* and N _0_ are the total biomasses (measured as optical density, OD) before and after the exponential growth phase, and ∆t is the duration of the exponential phase. Exponential phase was determined by conducting a series of windowed linear regressions over the full growth curve, and retaining the part of the curve with the largest slope (computer code given in Supplementary materials part 3).

Individual cell size was measured by flow-cytometry using a FacsCantoII (BD BioSciences, San Jose, California, USA), and data (forward scatter) were analysed using the *flowCore* package ^53^ in R 2.12.0 ^54^. Each measure was performed on a sample of 2×10 ^5^ cells without dyes.

Measures of OD will be affected by changes in particle size. At equal bacterial density, a population of smaller cells will yield a lower OD value, because fewer particles will block less of the incoming light. The practical conclusion is that whenever bacteria get smaller, we understimate their count, and thus their growth rate. Because this means that we are *more* conservative about the impact of phage exposure on growth rate (i.e., if there were any bias in our results, it would be an underestimation of the increase in growth rate), we did not correct for this effect.

We also estimated the sensitivity of the different treatments to live phage by measuring changes in bacterial populations. At each 24-hour transfer, 1% of the bacterial population was placed in 2 mL of fresh KB, and 20 µL of amplified phage ( *ca* 10 ^8^ viral particles) were added (a control without phage was conducted simultaneously). Bacteria CFUs were counted on solid agar after 48 hours of incubation to estimate population size.

Due to non-normality of the data as assessed by a Shapiro test, we used a Kruskal-Wallis test to determine the significance of the between-treatments effects.

## Supplementary materials

### 1. Molecular biology protocol and results

*PCR cycle* – 6 minutes at 95°C, then 30 cycles of 1 minute at 94°C, 1 minute at 55°C, 2 minutes at 72°C, then 10 minutes at 72°C.

*PCR buffer* – 5 μL of buffer, 4 μL of primers at 10 pM/mL (for both TPV1f and TPV1r), 2 μL of dNTP at 5 pM/mL, 1.5 μL of MgCl2 at 25 mM, 5.35 μL of H20, 3 μL of sample DNA, 0.15 μL of TaqPol - conducted with a GoTaq FlexiDNA Polymerase M8301 kit from Promega.

### 2. Preliminary experiments and phage

We verified the efficiency of the phage inactivation protocol by incubating the bacterial strain used for the main experiment with either live phage or phage exposed to UV for 2hrs or 4hrs. We measured the Malthusian fitness of 6 host populations near carrying capacity at low temperature (4°C, growth restrictive) over the course of 24hrs (the difference with the experiment presented in the main text is that inactivated phage *were not removed* over the course of this pilot study).

### 3. Determination of the maximal growth rate (R code)

givegrowth = function (y, x = c(1:length(y)), bw = 12)

## y : optical density

## x : times of the measures

## bw : number of points to include in regression

{

    list.of.coeff < - NULL

    for (i in 1:(length(x) - bw)) {

      part.x < - x[i:(i + bw)]

    part.y < - y[i:(i + bw)]

    cur.lm < - lm(part.y ~ part.x)$coeff[2]

    list.of.coeff[i] < -cur.lm

}

result < -max(list.of.coeff)

pos < -match(max(list.of.coeff), list.of.coeff)

coeff < -lm(y[pos:(pos + bw)] ~ x[pos:(pos + bw)])$coeff

return(as.numeric(coeff[2]))

}
