# Supplementary material for: Terminal investment induced by a bacteriophage in a rhizosphere bacterium
Source: F1000Res. 2013 May 20;1:21. Originally published 2012 Oct 2. [Version 2] doi: 10.12688/f1000research.1-21.v2 (PMC4964844; doi:10.12688/f1000research.1-21.v2)
Supplement: Peer review history

# Peer review: Referee response for version 2

**Published:** 2013-06-20

**Authors:** Britt Koskella

**Affiliations:** 1 Centre for Ecology & Conservation Biosciences, College of Life & Environmental Sciences, University of Exeter, Exeter, UK

Poisot and coauthors have now discussed in more detail the potential mechanisms underlying the intriguing result they have uncovered. They have also emphasised that the result is robust against the problems associated with examining population growth of cells of differing size using optical density readings. It will indeed be interesting in the future to determine the coevolutionary implications of such rapid phenotypic change despite no obvious fitness benefit (i.e. no increased resistance to phage). 

**I have only one minor comment remaining:**

 Perhaps I am missing something, but the legend for figure 2 states that the variable presented is the forward scatter and yet the paragraph below states that there were no observed differences for forward scatter, only side scatter. Please could you clarify?

I have read this submission. I believe that I have an appropriate level of expertise to confirm that it is of an acceptable scientific standard.

# Response

**Published:** 2013-06-30

**Authors:** Timothée Poisot

**Affiliations:** Université du Québec à Rimouski, Canada

We apologize for the mistake. The error is in the text, in which we inverted the parameters. We will see with the editorial office that this mistake is corrected.

# Peer review: Referee response for version 2

**Published:** 2013-06-11

**Authors:** Paul Turner

**Affiliations:** 1 Department of Ecology and Evolutionary Biology, Yale University, New Haven, CT, USA

The authors provided greater clarity on the controls for their study, and this was my main concern with the earlier submission. Although the mechanistic details on phage exposure and its apparent effects on bacterial growth have yet to be completely elucidated, the phenomena in this study are highly interesting and readers will be intrigued by these results.

I have read this submission. I believe that I have an appropriate level of expertise to confirm that it is of an acceptable scientific standard.

# Peer review: Referee response for version 1

**Published:** 2012-10-22

**Authors:** Paul Turner

**Affiliations:** 1 Department of Ecology and Evolutionary Biology, Yale University, New Haven, CT, USA

**1.** ls there a subpopulation of the UV inactivated phage that are destroyed in the process of creating them, such that addition to bacterial cultures might constitute addition of DNA that can be taken up through transformation ? If so, is it possible that this transformed DNA is being used as a nutrient by the bacteria? This might explain the slight increase in growth rate of phage-exposed bacteria in the experiments. There is precedent in other bacterial systems, but I do not know whether this provides an alternative explanation in the current study. The authors should take this possibility into account.

**2.** It is unclear what constitutes the controls performed in this study. One choice of control would be to obtain the UV-inactivated phage, and then remove these particles via centrifugation. The particle-free supernatant would then be added to controls, so that all components (except phage presence) would be otherwise identical across treatments and controls. However, it is unclear whether this was the approach used, and therefore I am worried that the chosen control is insufficient for drawing proper conclusions in the work.

Overall, the work seems very preliminary, and the data presented are not strongly supportive of the conclusions drawn.

I have read this submission. I believe that I have an appropriate level of expertise to confirm that it is of an acceptable scientific standard, however I have significant reservations, as outlined above.

# Peer review: Referee response for version 1

**Published:** 2012-10-08

**Authors:** Britt Koskella

**Affiliations:** 1 Centre for Ecology & Conservation Biosciences, College of Life & Environmental Sciences, University of Exeter, Exeter, UK

Understanding the response of bacterial populations to bacteriophage viruses is of central importance to predicting microbial dynamics. To do so requires knowledge about both the ecological and evolutionary responses of bacteria to phage in the environment.

This includes possible changes in bacterial growth rate and cell size, as both have been shown to affect the rate of adsorption of phages by host cells (e.g. Hadas *et al.* 1997). In this paper, Poisot and coauthors investigate the inducible response of bacteria to phages by using UV-treated phages that are capable of binding to, but not infecting their host cells. They find that bacteria encountering UV-treated phages have a faster doubling time and smaller cell size than the control bacterial populations but that this response is short-lived and does not confer resistance to the phage.

This is a very intriguing result that confirms work from studies using live phages (e.g. Gómez, P. and A. Buckling. 2011) and suggests that binding of phages, regardless of subsequent infection success, might play a key role in shaping bacterial population dynamics. The approach taken is a very nice way to look for inducible responses to phage and the results are, for the most part, very clear. I do, however, wonder about the independence of the results for doubling time (measured as optical density) and cell size. Surely the optical density measure is affected by the cell size? The authors state that “Analyses of the distribution of several flow cytometry profiles showed that a difference in cell shape is unlikely to explain this result (see data associated to this article)” Since this is an absolutely central result of the finding, I would find it very helpful if the authors actually presented and discussed this evidence. In fact, it was not clear to me which data were in support of this. Otherwise, I do not think that the results should be used to primarily suggest a phage-induced response of increased growth rate, with a cost of decreased cell size. Instead, perhaps it is a response of decreased cell size with a subsequent small change in doubling time? I imagine the authors have the analyses to rule out the latter possibility. Further to this, when the authors do look directly at colony forming units, rather than optical density, in their analyses of bacterial resistance to live phages they do not find a difference among the treatments. In this case, when bacteria were exposed to live phages, bacterial populations that had been exposed to inactivated phages grew to the same densities over 24 hours as those that had not been exposed to inactivated phages. I find this hard to interpret as it could suggest that a) the previous results were primarily indicative of a change in cell size, rather than growth rate, or even that b) the bacteria from the inactivated phage treatments do have a higher growth rate but were more susceptible to phages and thus had the same CFU. It would be helpful if the authors could discuss this result in more detail.

I have a few additional points of clarification that I think would help readers fully understand the results.

First, I wonder whether the authors could clarify their thoughts on the mechanism underlying the change to smaller cell size and/or increased doubling time of bacteria encountering inactivated phages. For example, could it be that small cell size is a response to altered numbers or activity of receptors on the bacterial cell surface? It seems that phage binding to receptors could alter their function and thus those bacterial cells with bound inactivated phages could be smaller due to decreased uptake of resources.

Second, I think the finding that bacteria treated with inactivated phages show changes in growth rate and/or cell size but do not differ in terms of their resistance to live phages is quite interesting! I would be keen to know what the infection rates of the control and treated populations were, as this would help with interpretation of the result. It seems surprising that there is no change in resistance, as previous evidence suggests a strong correlation between cell size and growth rate with adsorption rate. Might this result give insight to the mechanism underlying the changes observed? The authors mention that smaller surface area would mean a lower encounter rate with phages, but this doesn’t seem to be the case when the cells are exposed to live phages.

As a very minor point, I wonder whether the authors meant to say that bacteria were separated from unbound, rather than bound, phages in their methods section, as it is unclear how centrifugation would separate bacteria from bound phages.

I have read this submission. I believe that I have an appropriate level of expertise to confirm that it is of an acceptable scientific standard, however I have significant reservations, as outlined above.
